# Pathophysiological Relationship between Infections and Systemic Vasculitis

**DOI:** 10.1155/2015/286783

**Published:** 2015-07-07

**Authors:** Carolina Muñoz-Grajales, Juan C. Pineda

**Affiliations:** ^1^Rheumatology Department, Pablo Tobón Uribe Hospital, Medellín, Colombia; ^2^Internal Medicine Department, Fundación Valle del Lili, ICESI University, Cali, Colombia

## Abstract

The development of autoimmune disorders requires a combination of genetic, immunological, and environmental factors. Infectious agents, such as viruses and bacteria, can trigger autoimmunity through different mechanisms, and for systemic vasculitis in particular, microbial agents have been suggested to be involved in its pathogenesis. Although the exact mechanisms have not been fully elucidated, different theories have been postulated. This review considers the role of infections in the etiology of primary vasculitis, emphasizing their related immunological events.

## 1. Introduction

Primary systemic vasculitides are a complex group of disorders that involve multiple organs, often with a severe clinical course. Although uncommon, they require prompt management and adequate handling. Early diagnosis is required to preserve the function of the affected organs and to decrease mortality. Clinical manifestations are varied and nonspecific and may be related to those occurring in other systemic diseases, such as infections or malignancies. Although the origin of primary vasculitis is not fully understood, there is a combination of genetic, immunological, and environmental factors, like infections, which could trigger some of them. In the latest revision of the Chapel Hill Consensus Conference (CHCC), vasculitis associated with hepatitis B (HBV) and hepatitis C (HCV) viruses were included [1]; however, there are many other organisms associated (Table 1). Since Wegener's first description, an association with bacteria [2] has been suspected. There have been attempts to confirm this relationship; however, it remains a subject of debate.

Various mechanisms have been proposed to be involved in the relationship between infection and systemic vasculitis: molecular mimicry, superantigens, cell activation by Toll-like receptors (TLR), and more recently, the anti-idiotypic response by neutrophil extracellular traps in the antineutrophil cytoplasmic antibodies- (ANCA-) associated vasculitis (Table 2) [3–5]. In addition, some infections are accepted as the secondary causes of vasculitis. Bacteria, spirochetes, rickettsia, fungi, viruses, parasites, and protozoa have all been implicated, and various mechanisms have been postulated, including direct vascular invasion, immune complex deposition, and activation of T lymphocytes [6–8]. In this paper, we review the current evidence for the role of infections in the etiology of primary vasculitis. We emphasize immunological events and their relationships to organ damage.

## 2. Large and Medium-Vessel Vasculitis

For several years, a causal link between Takayasu's arteritis (TA) and tuberculosis (TB) has been proposed due to the granulomatous nature of both diseases. Moreover, the link is supported by the increasing frequency of TA in patients from continents with a high prevalence of TB, such as Asia, Africa, and South America [5]. However, attempts to recover mycobacteria from the tissues of TA patients have been unsuccessful. In 2012, Soto et al. found sequences of the* Mycobacterium tuberculosis* IS 6110 and HupB genes in the aorta of 23 of 33 patients with TA (70% of TA tissues compared with 32% of atherosclerotic tissues) [9]. Furthermore, it has been observed that patients with TA have an increased T cell response to the human heat shock protein 60 (hHSP-60) and mycobacteria heat shock protein 65 (mHSP-65) (22 of 26 patients versus 3 of 18 healthy controls). Additionally, an increase in circulating IgG isotype antibodies against these antigens was reported (anti-MHSP-65 in 92% of TA patients versus 11% in controls and anti-hHSP-60 in 84% of TA subjects versus 22% in controls; *P* < 0.001), raising the possibility of molecular mimicry between hHSP-60 and hHSP-65 [10]. Therefore, a previous infection with TB could aid in the development of arteritis because of the reactivity against vascular endothelial peptides with similarity to mycobacterial antigens [37].

In giant cell arteritis (GCA), the presence of certain viruses in the tissues of temporal arteries from affected patients has been suggested. However, a study conducted on 37 samples to detect* Chlamydia pneumoniae*, parvovirus B19, and herpesvirus (excluding serotype 8) showed no differences between the patients with GCA and controls. Nonetheless, Mohammadi et al. found human papilloma virus DNA in 16 of 22 temporal artery samples from patients with GCA vasculitis [50] and Curly et al. reported* Burkholderia* sp. in 9 out of 10 patients [11]. However, a recent microbiome DNA sequencing study in samples from the temporal arteries of patients with GCA showed no increase in the frequency of a particular microorganism [51].

In contrast, polyarteritis nodosa (PAN) is strongly linked to hepatitis B virus (HBV) and also has been linked to hepatitis C virus (HCV), human immunodeficiency virus (HIV), group A* Streptococcus*, human T cell leukemia virus-1,* Cytomegalovirus*,* Epstein-Barr virus*, and parvovirus B19 [12]. Thus, the prevalence of PAN associated with HBV, HCV, and HIV coincides largely with the prevalence of underlying infections [5].

The association between PAN and HBV has been frequently observed (10–54% of patients); in fact, in the last CHCC, this association was grouped in a specific subset of vasculitis called vasculitis associated with probable cause [1]. The vasculitis typically begin within the first 6 months after infection when there is constant viral replication and immune complex deposition composed of viral antigens, more likely HBe antigen, and other specific antibodies [3]. All cases of HBV-PAN are almost always associated with the wild-type HBV infection, characterized by HBe antigenemia and high HBV replication. Whereby, two general mechanisms have been proposed, the first one is a direct injury of the vessel wall by the virus and the second one an endothelial lesion resulting from circulating immune complexes deposition and complement activation, leading to attraction and activation of neutrophils. That could explain the subsequent organ damage [12] but still need further research to confirm this.

## 3. Small-Vessel Vasculitis

### 3.1. Immune Complex-Mediated Vasculitis

Regarding basement membrane disease, only a description of infection (influenza outbreak) is thought to be a likely trigger [13]. In contrast, the relationship between HCV and cryoglobulinemia is well known. Although the exact mechanism is unknown, it has been suggested that the virus induces B cell proliferation via the interaction of its envelope E2 protein with CD81 present in hepatocytes and lymphocytes, thereby generating chronic B lymphocyte stimulation [14–16]. Additionally, although the cause of IgA-mediated vasculitis is unknown, some infectious triggers have been identified. One study reported that 29 of 55 children [17, 18] were isolated with* Helicobacter pylori* (49.3% of Chinese infants with IgA vasculitis compared to 23.4% of controls) [19, 20].* Staphylococcus aureus* [21] and* Mycoplasma pneumoniae* [22] are additional examples of these associations. Similarly, hypocomplementemic urticarial vasculitis syndrome, an illness that has recently been considered a monogenic form of lupus by some researchers [52], does not have many descriptions related to infection. In a study of 64 patients published in 2009, 70% had no identifiable cause of vasculitis. In only 12% of the cases, infection (primarily of the upper respiratory tract) was considered to be the underlying cause [23].

### 3.2. ANCA-Associated Vasculitis (AAV)

Vasculitis associated with antineutrophil cytoplasmic antibodies (ANCA) includes a group of diseases that cause small-vessel necrosis. These diseases are characterized by the presence of ANCA which vary depending on the vasculitis type and whether they are directed against myeloperoxidase (MPO) or proteinase 3 (PR3) [53, 54]. Some researchers propose that PR-3 ANCA-associated vasculitis patients are more likely to relapse and display predominant extrarenal manifestations and rapid deterioration of renal function compared to patients with MPO-ANCA vasculitis [55–57]. Genetic factors are also different, because PR3-ANCA vasculitis is partnered with HLA-DP and the genes encoding PR3 (PRTN3) and antitrypsin (SERPINA1). In contrast, MPO-ANCA vasculitis is associated with HLA-DQ [58, 59].

Currently, there is only one* in vivo* model for MPO-ANCA vasculitis, but not for the associated PR3. Immunizing MPO-deficient mice produced anti-MPO. The transfer of splenocytes from immunodeficient* Rag2* ^−/−^ mice induced necrotizing vasculitis with granulomatous inflammation and pauci-immune glomerulonephritis. Injecting IgG into wild immunodeficient MPO mice also generated a pauci-immune glomerulonephritis [60, 61]. Although immunized mice developed antibodies capable of recognizing PR3 on the surface of neutrophils, the passive transfer of IgG did not lead to vasculitis. Immunizing mice with the human PR3 ANCA produced some of the inflammatory reactions in the lungs, but not glomerulonephritis [62–64]. These differences suggest that although ANCA production is shared between these vasculitides (MPO-ANCA and PR3-ANCA), they have different pathogenic pathways [65] and as discussed below, iPR3-AAV has the most notable microorganism participation in its pathophysiology.

Neutrophil extracellular traps (NETs) have recently been reported to participate in the pathogenesis of various autoimmune diseases, such as AAV (MPO-ANCA and PR3-ANCA vasculitides). Immunofluorescence analyses of NETs induced* in vitro* revealed that both MPO and PR3 are expressed in extracellular chromatin fibers [38, 39]. These authors also found that IgG ANCA induced NETs in the patients with AAV; similarly, it was also observed that murine anti-PR3 could induce NET formation [38]. In serum samples of crescentic glomerulonephritis lesions associated with AAV, increased NETs and DNA-MPO were detected [38]. Furthermore, it has been reported that propylthiouracil (PTU) can generate anti-MPO-ANCA, and some patients may develop ANCA-associated vasculitis. Nakazawa et al. studied the capacity of PTU to induce aberrant or irregular NETs* in vitro*. Typically, NETs are obtained after the* in vitro* stimulation with phorbol myristate acetate (PMA). However, these authors added PTU to PMA, resulting in the production of irregular NETs. These were subsequently inserted into WKY rats and they produced ANCA, which differed from the rats immunized with regular NETs produced by PMA. Pauci-immune glomerulonephritis and alveolar hemorrhage have also been induced in rats exposed to PTU/PMA [40].

For several years, the involvement of* Staphylococcus aureus* infection in the genesis of Wegener granulomatosis (GPA) has been suggested. This theory emerged based on the observation that up to 70% of patients with chronic nasal GPA are carriers of this microorganism [25–30]. Moreover, there is an increased risk of relapse of the limited form in carriers [25, 31], and trimethoprim-sulfamethoxazole prophylaxis reduces the risk of recurrence by up to 60% [32, 33]. Furthermore, a recent retrospective study found that 70% of patients with airway GPA treated with trimethoprim-sulfamethoxazole monotherapy had good clinical responses [34]. Finally, in the microscopic polyangiitis and IgA vasculitis, there are case reports of activation after* S. aureus* infections [24, 35].

Different theories have been developed regarding the relationship between GPA and* S. aureus*. One theory proposes that carriers of the bacteria have low-grade infections in the upper respiratory tract, resulting in the “priming” of neutrophils induced by cell surface PR3 expression, which can be activated by PR3-ANCA. The possibility of polyclonal expansion of B cells, stimulated by the components of the bacterial cell wall, has also been considered. Others have suggested a chronic stimulation of T cells by* S. aureus* superantigens, because they found persistent activation of circulating T lymphocytes in patients with GPA [28, 36, 66, 67].

One study found that, in GPA chronic carrier patients of* S. aureus*, strains expressing toxic shock syndrome toxin 1 (SAT-1) had the increased numbers of relapses (relative risk versus control subjects 13.3; 95% CI: 4.2–42.6) [27]. However, in another study, these same authors reported that although they observed further T lymphocyte expansion in GPA, they found no association with the presence of* S. aureus* or superantigens [7]. Another hypothesis assumes that the antigens of these bacteria can adhere to the renal basement membrane through interaction with the electric charge [41].

More recently, another mechanism based on the similarity of some bacterial peptides was proposed. It includes (but is not limited to)* S. aureus* with the complementary PR3 protein (PR3c) produced in the bacteria by antisense or reverse translation of the* PRTN3* gene, which encodes human PR3, into cDNA that is experimentally incorporated into bacterial DNA [68]. These theories resulted from the observation that seven of 34 patients with PR3-ANCA had antibodies against PR3c (based on ELISA against the PR3c middle region) but had no cross-reactivity with PR3. Therefore, anti-PR3 could be bonded against anti-PR3c, indicating an idiotype anti-idiotype reaction [42].

Because the aforementioned bacterial peptides have similarities with PR3c, GPA carriers of* S. aureus* can theoretically produce antibodies against PR3c through the idiotypic anti-idiotypic reactions equivalent to PR-3 ANCA [42]. In support of this theory, mice immunized with PR3c increased their anti-PR3 antibody production. However, in a subsequent study of 57 Dutch patients with PR3 AAV and 27 healthy controls, no increase in reaction against the middle region of PR3c or any relationship between* S. aureus* was noted [69]. Because IgG subclass affects binding affinity, these contradicting results could be explained by the differences in the profile of IgG subclasses within the populations studied, as suggested by Preston and Falk [70]. Alternatively, perhaps anti-idiotype antibodies may mask or hide the antigen-binding site in the idiotype, preventing binding to the complementary protein used in ELISA assay. This is analogous to observations in a study of anti-idiotype SSA/Ro in patients with systemic lupus erythematosus or Sjögren syndrome [70, 71].

In AAV, TLR expression on monocytes and natural killer cells in peripheral blood [43] was observed to be an important element in the response to infection by innate immunity; therefore, another theory postulates that the activation of neutrophils in patients with AAV is related to TLRs [67]. In a cohort of patients with GPA, the expression of TLR2, TLR4, and TLR9 on granulocytes was not increased in uninfected patients compared to healthy controls, even when the disease was active. However, during active infection, there was a significant increase in the TLR9 expression, compared to uninfected patients; there was also a tendency towards an increased TLR2 and TLR4 expression [44]. Another study found an increased expression of TLR9 in monocytes of* S. aureus* carriers with AAV [43]. In addition, when TLR9 and TLR2 were stimulated* in vitro*, they increased the expression of membrane PR3 (PR3m) [44]. Moreover, it was reported that in patients with PR3-AAV, IL-2 in combination with hypomethylated CpG motifs, which are characteristic of viruses and bacteria, triggered the production of PR3-ANCA by autoreactive B cells [45, 67].

Finally, another important finding, which supports the relationship between AAV and bacterial infection, is the presence of human autoantibodies against protein 2 of the lysosomal membrane (hLAMP-2) in patients with AAV (80% of patients from three European cohorts) [46]. These antibodies activate neutrophils and generate endothelial damage* in vitro* [47] and have homology with the bacterial adhesion FimH protein (a fimbrial adhesin of Gram-negative bacteria). Eight amino acids of an epitope of LAMP-2 (P41–49), which are recognized by the autoantibodies, have a strong homology to several FimH proteins that are common in Gram-negative species, thereby suggesting molecular mimicry between the two proteins [66]. In* in vivo* models, immunization of rats with FimH generates reactive antibodies, LAMP-2, and necrotizing pauci-immune nephritis [47, 48]. However, in other cohorts, an increase in the anti-hLAMP-2 has not been observed [49].

Other organisms, such as* Haemophilus influenzae*,* Streptococcus pneumonia*, and* Klebsiella pneumoniae*, have been found in the lower respiratory tract and have been considered part of the etiology of GPA [72]; however, more evidence is needed. In addition, antibodies against HCV and* Helicobacter pylori* also have been found [73]. Other viruses involved in the onset of AAV include HIV [74] and Epstein-Barr virus (EBV). EBV functionally inactivates a protein expressed primarily in T cells, thus altering the immune response and resulting in systemic vasculitis and lymphocyte proliferation through the mitogenic effect of viral DNA [75, 76]. Cytomegalovirus produces direct cellular damage and induces high plasma levels of immune amplifiers, such as IL-5 and *α*-lymphotoxin [77].

## 4. Conclusions

Most infectious agents, including viruses, bacteria, and parasites, can trigger autoimmunity through various mechanisms. Infections are associated with the secondary forms of vasculitis. However, there is growing evidence that microbial agents also play a role in the induction of primary systemic vasculitis. For example, a number of infectious agents have been proposed as potential triggers; however, in most cases, the link remains hypothetical. Different mechanisms and pathogenic hypotheses have been proposed (molecular mimicry, superantigens, cell activation by Toll-like receptors, the anti-idiotypic response, and neutrophil extracellular traps); however, the pathophysiology of the complex relationship between infection and vasculitis remains not fully understood.

## Figures and Tables

**Table 1:** The most important microbial agents presumed to be involved in the development of primary systemic vasculitis.

	Microbial agent	Reference
Type of vasculitis		
Takayasu arteritis	*Mycobacterium tuberculosis *	[5, 9, 10]
Giant cell arteritis	*Burkholderia *	[11]
Polyarteritis nodosa	Hepatitis B virus, hepatitis C virus, and HIV infection	[12]

Immune complex vasculitis		
Antiglomerular basement membrane disease	Increased incidence during influenza epidemics	[13]
Cryoglobulinemic vasculitis	HCV	[14–16]
IgA vasculitis	Many bacteria, viruses, and even protozoa (e.g., *Helicobacter pylori*, *Staphylococcus aureus*, and* M. pneumonia*)	[17–22]
Hypocomplementemic urticarial vasculitis	Few associations with infections	[23]

ANCA-associated vasculitis		
Microscopic polyangiitis	*Staphylococcus aureus *	[24]
Granulomatosis with polyangiitis	*Staphylococcus aureus,* *Klebsiella,* and *Escherichia coli* species	[24–36]
Eosinophilic granulomatosis with polyangiitis	—	

**Table 2:** Theories regarding the role of infection in the development of primary systemic vasculitis. Toll-like receptor (TLR); 3 proteinase (PR3); myeloperoxidase (MPO).

Vasculitis	Mechanism	Microbial agent	Evidence	References
Takayasu's arteritis	Molecular mimicry	*Mycobacterium tuberculosis *	*Mycobacterium tuberculosis* gene sequences IS6110 and HupB in tissues from aorta Increased T cell response to hHSP60 and mHSP65	[9, 10, 37]

MPO-AAV	Neutrophil cell traps	Various	Immunofluorescence analyses revealed MPO located in these extracellular chromatin fibers IgG ANCA induced neutrophil NETs In crescentic glomerulonephritis, lesion associated with AAV has detected NETs Increased levels of MPO-DNA complex in the serum Induced pauci-immune glomerulonephritis and alveolar hemorrhage in rats exposed to PTU/PMA	[38–40]

PR3-AAV	Neutrophil cell traps	Various	Immunofluorescence analyses revealed MPO located in these extracellular chromatin fibers Murine anti-PR3 antibody induced NETs	[38]
	Stimulation of T lymphocytes by *S. aureus* superantigen	*S. aureus *	Persistent activation of circulating T lymphocytes Chronic carriers of *S. aureus* that expressed toxic shock toxin 1 (TSS-1) had increased numbers of relapses	[27, 28, 36]
	Antigens can join to renal basement membranes by charge interaction	*S. aureus *	—	[41]
	Idiotype, anti-idiotype	*S. aureus *	Similarity of some bacterial peptides, including but not limited to *S. aureus*, with complementary PR3 (PR3c)	[42]
	TLR activation	*S. aureus *	During active infection of AAV, TLR9 expression increased significantly compared to uninfected patients Increased expression of TLR9 in monocytes from patients with AAV (*S*. *aureus* carriers) TLR2 and TLR9 were stimulated *in vitro*, and increased membrane expression of PR3 (PR3m) In patients with AAV-PR3, hypomethylated CpG motifs triggered production of PR3-ANCA by autoreactive LB	[43–45]
	Molecular mimicry	*Klebsiella* and *Escherichia coli* species	Anti-lysosomal-associated membrane protein-2 (hLAMP-2)	[46–49]
